# Effect of Laryngeal Mask Airway Insertion on Parameters Derived From Catacrotic Phase of Photoplethysmography Under Different Concentrations of Remifentanil

**DOI:** 10.1109/JTEHM.2020.3017368

**Published:** 2020-08-17

**Authors:** Wanlin Chen, Ying Feng, Xinzhong Chen, Feng Jiang, Jiajun Miao, Shali Chen, Hang Chen

**Affiliations:** 1 College of Biomedical Engineering and Instrument ScienceZhejiang University 12377 Hangzhou310027China; 2 Zhejiang Provincial Key Laboratory of Cardio-Cerebral Vascular Detection Technology and Medicinal Effectiveness AppraisalHangzhou310027China; 3 Department of AnesthesiaWomen’s Hospital, School of MedicineZhejiang University 12377 Hangzhou310006China; 4 Zhejiang LaboratoryConnected Healthcare Big Data Research CenterHangzhou311121China

**Keywords:** Balance of nociception-anti-nociception, catacrotic phase, photoplethysmography, wavelet de-noising

## Abstract

Background: Some parameters have been extracted from photoplethysmography (PPG) with a good relativity with nociception, but without encouraging results in qualifying the balance of nociception-anti-nociception (NAN). The features of PPG have not been thoroughly depicted and more prospective univariate parameters deserve to be explored. The aim of this study was to investigate the ability of parameters derived from catacrotic phase of PPG to grade the level of analgesia. Methods: 45 patients with ASA I or II were randomized to receive a remifentanil effect-compartment target controlled infusion (Ce_remi_) of 0, 1, or 3 ng/ml, and a propofol effect-compartment target controlled infusion to maintain an acceptable level of hypnosis with state entropy (SE) at 40~60. Laryngeal mask airway (LMA) insertion was applied as a noxious stimulus. Five diastole-related parameters, namely diastolic interval (DI), diastolic slope (DS), the minimum slope during catacrotic phase (DSmin), the interval between DSmin and its nearest trough (DTI), and area difference ratio (ADR), were extracted. Pulse beat interval (PBI) was calculated as a reference parameter. Results: LMA insertion elicited a significant variation in all parameters except ADR during Ce_remi_ of 0 and 1 ng/ml. Compared to PBI (prediction probability ($\text{P}_{\text {K}}$) = 0.796), the parameters of DI, DS, and DTI presented a better consistence with the level of anti-nociceptive medication, with $\text{P}_{\text {K}}$ of 0.825, 0.822, and 0.822 respectively. Conclusion: The features extracted from catacrotic phase of PPG, including DI, DS, and DTI, could provide a promising potential to qualify the balance of NAN.

## I. Introduction

The assessment of the adequacy of analgesia, namely the balance of nociception-anti-nociception (NAN), has received wide attention in recent years. An accurate quantification of the balance of NAN is critical in peri-operative care because over- and underdosage of analgesic drugs could cause immunologic imbalance and post-operative hyperalgesia respectively, which significantly affect the outcome of patients [1]–[3]. Hemodynamic stability, such as changes in heart rate and blood pressure, and clinical signs like sweating and tearing are routinely suggested as indicators of the occurrence of nociceptive event. Moreover, several surrogate indices have been commercialized to guide the administration of analgesics more automatically in clinical practice [4]. Surgical pleth index (SPI), analgesia nociception index (ANI), and the nociception level (NoL), three popular commercial indices developed from photoplethysmography (PPG), electrocardiography (ECG), and skin conductance (SC), have been shown to react better to noxious stimulus than standard hemodynamics [5]–[7]. Nonetheless, controversial results about the capability to predict inadequate balance of NAN and the influence on postoperative condition are existed as well [8]–[12]. Hence, a more reliable index is desirable. Based on the peculiarities of the available indices, two approaches we are able to try for improving the feasibility. For one thing dig out more subtle nociception-triggered changes hidden in signals. The physiological state of patients could be directly reflected in the morphology of signals, therefore, the variations associated with analgesia could be captured by specific features. More relevant and detailed features are required as they form the basis for the development of multiparametric indices. For another, optimize the algorithm to integrate univariate parameters into a multivariate index when the multiparameter is considered to be more robust against confounding factors. This study will focus on the former.

PPG, a non-invasively obtained signal, offers a relatively easy access to the assessment of peripheral vasoconstriction (sympathetically mediated) via finger probe without other consumables. With the advantages of easily-operated data acquisition and simple morphology, PPG has been widely analyzed with respect to the correlation with the balance of NAN. Some features, inclusive of PPG systolic peak amplitude, pulse beat interval (PBI), rising slope, systolic interval, the difference of amplitude between two adjacent systolic peak points, and the difference of amplitude between two diastolic peak points within a waveform, have been extracted in time domain with a good relativity with nociception, but without encouraging evaluation results about their capacity in qualifying the balance of NAN [13]–[15]. This indicates that although the extraction of the target information from PPG is promising, the features of PPG have not been thoroughly depicted and more prospective univariate parameters deserve to be explored.

Morphologically, PPG signal could be divided into two phases, anacrotic phase and catacrotic phase. Anacrotic phase is the rising edge of signal and catacrotic phase is the falling edge, signifying systolic and diastolic period respectively. Catacrotic phase which contains the message of retrograde flow resulted from aortic valve closure is deemed to have richer features with its more complex shape. Thus, this study mainly paid attention to the details of catacrotic phase of PPG.

In this study, five parameters were derived from catacrotic phase of PPG. The objective was to investigate the ability of parameters to grade the level of analgesia during a constant hypnotic level, expecting that (1) parameters have a significant reaction to stimulus with no or low analgesics administration; (2) parameters change monotonically with the increasing analgesic doses after stimulation occurred.

## II. Material and Methods

### A. Study Population

With the approval of the Ethics Committee of Women’s Hospital, School of Medicine, Zhejiang University (No. 20170131) and written informed consent, 45 female patients scheduled for gynecologic laparoscopy or breast surgery under general anesthesia, American Society of Anesthesiologists (ASA) physical status I or II, were enrolled. Exclusion criteria include body mass index (BMI) over 30 kg/$\text{m}^{2}$, difficult airway (Mallampattis test III or IV), chronic use of psychoactive medication or abuse of alcohol, and a history of cardiovascular disease or autonomic nerve dysfunction.

The randomization was performed to allocate patients into three groups using Excel (version 2010, Microsoft Corporation, Redmond, WA, USA). First, in a table of Excel with a list of patients’ names, a list of random numbers was generated using RAND function. Then the whole table was sorted in ascending order using the list filled with random numbers, and serial number was marked correspondingly. Based on the remainder of serial number divided by three, patients were randomized to receive one of three different remifentanil effect-compartment concentrations (Ce$_{\mathrm {remi}}$) infusion during anesthesia: 0 (n = 15, R0), 1 (n = 15, R1), and 3 (n = 15, R3) ng/ml. It should be noted that the randomization was not completed once and each randomization was executed in patients with similar scheduled time for surgery. To assign patients equally to each group, the number of patients for each randomization was set as a multiple of three.

### B. Anesthesia Protocol and Data Collection

Anesthesia was supervised by an experienced staff anesthesiologist in all cases.

Patients received no premedication. Induction of anesthesia was administrated via a target-controlled infusion (TCI) (Orchestra Primea, Fresenius, France) of propofol and remifentanil, with pharmacokinetic models introduced by Schnider et al and Minto et al respectively [16], [17]. A propofol infusion was adjusted to achieve and maintain an acceptable level of hypnosis with state entropy (SE) at 40~60, followed by remifentanil infusion with target Ce_remi_ steering. An infusion of 0.15 mg/kg cisatracurium was performed to facilitate laryngeal mask airway (LMA) insertion which was applied as a noxious stimulus. During the period of 3 min before and after stimulus, the hypnotic level and remifentanil concentration were kept at a steady state for data collection. The time stamp of LMA insertion was annotated manually.

**FIGURE 1.**
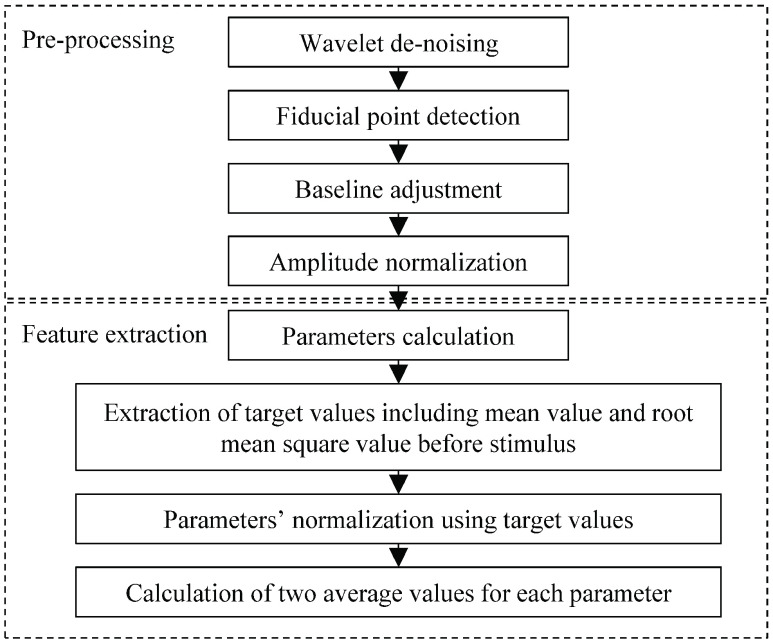
Process flow diagram for data pre-processing and feature extraction.

PPG signals from the index finger of the right hand were continuously recorded using anesthesia monitoring (CARESCAPE B650, GE Healthcare, Finland) with an Fs = 100 Hz sampling frequency. Data of 60 s before and after LMA insertion were selected and analyzed off-line using Matlab software (version R2017b, Mathwork Inc., MA, USA). Given that it is difficult to manually mark the event within seconds when it actually occurs, the data of 10 s before and after stimulus were discarded. Consequently, the data within the window of −70 to −10 s before stimulus and +10 to +70 s after stimulus were designated for analysis. Process flow diagram for data pre-processing and feature extraction is shown in Fig. 1.

### C. Data Pre-Processing

#### 1) Wavelet De-Noising

In order to acquire clean noise-free PPG data preserving the essential morphological features, discrete wavelet transform (DWT) was applied to the raw signals.

Fourier transform is a commonly used tool to filter out mixed noise through removing a specific frequency band. However, it does not work well when the signal is nonstationary [18]. The wavelet transform has the characteristics of multi-resolution analysis with the capability to extract both time and frequency information from the signal [19], which is suitable for de-noising nonstationary physiological signals like PPG with stimulus-induced disturbances.

**FIGURE 2.**
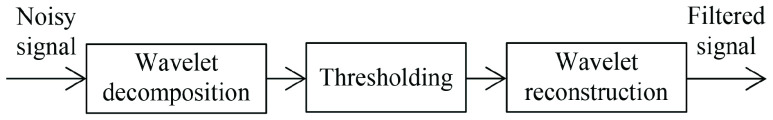
Wavelet de-noising procedure.

The steps of noise reduction method based on wavelet transform are clarified in Fig. 2 [20]. Initially, with the selected base wavelet, the noisy signal is decomposed to different wavelet coefficients including approximation coefficients cA(n) and the detail coefficients cD(n) gained after applying low pass filter (25/n Hz) and high pass filter (25/n Hz) respectively. Then a reasonable threshold value and a threshold function are deployed for separating the wavelet coefficients of signal from the wavelet coefficients of noise to achieve effective filtering. The filtered signal is eventually attained using inverse wavelet transform. According to the procedure of wavelet thresholding filtering above, four factors inclusive of base wavelet, wavelet decomposition layer, threshold determination method, and threshold processing function selection primarily affect the efficiency.

The selections of four factors were analyzed as follows. Since the frequency of cardiac portion contained in PPG signal ranges from 0.5 to 4 Hz [21], the number of decomposition layer was set as 3 to get a proper frequency range of approximation component in the last level. The frequency distribution bands of the extracted high frequency component and low frequency component in each layer are displayed in Table 1. Daubechies wavelet (db4) was chosen as base wavelet due to its superior performance [18].

**TABLE 1:** The Frequency Range of Each Frequency Component

Level n	1	2	3
cA(n); Hz	0–25	0–12.5	0–6.25
cD(n); Hz	25–50	12.5–25	6.25–12.5

For establishing the optimal threshold, the diverse combinations of two threshold functions and two typical thresholds were estimated using signal-to-noise ratio (SNR) and root mean square error (RMSE) calculated by formulas (1) and (2) respectively. Two threshold processing functions were soft and hard threshold function whereas the threshold values were determined by Principle of Stein’s Unibiased Risk (SURE) and Minimax thresholding. A single estimation of level noise based on first level coefficients was used for rescaling. A piece of PPG data was arbitrarily selected, and the performances of noise reduction are demonstrated in Table 2. The combination providing the highest SNR and lowest RMSE was chosen.

\begin{align*} SNR(dB)=&10\log _{10} \left [{ {\frac {\sum \limits _{i=1}^{n} {(x_{i} -\overline x)^{2}}}{\sum \limits _{i=1}^{n} {(y_{i} -x_{i})^{2}}}} }\right], \tag{1}\\ RMSE=&\sqrt {\frac {\sum \limits _{i=1}^{n} {(y_{i} -x_{i})^{2}}}{n}},\tag{2}\end{align*}

where $x_{i}$ is the original signal, $y_{i}$ is the filtered signal, and $n$ is the length of the signal.

**TABLE 2:** Performances of Signal Reduction After Thresholding Processing Using Different Threshold Functions and Threshold Determination Methods

Threshold determination methods	Principle of Stein’s Unibiased Risk		Minimax thresholding	
Threshold functions	Soft	Hard	Soft	Hard
SNR	56.4971	57.7061	48.3197	53.2511
RMSE	0.2377	0.2068	0.6093	0.3453

#### 2) Fiducial Point Detection

Systolic peak, dicrotic notch, diastolic peak, and trough are four major fiducial points of PPG. Affected by the position where finger clip sensor places and the variability of local blood volume, dicrotic notch and diastolic peak are not always visible. Taking into account the fact that the dicrotic notch and diastolic peak in most of PPG waveforms collected in this study are missing, the parameters related to these two points were not considered to be extracted and we merely operated the detection of systolic peak and trough.

**FIGURE 3.**
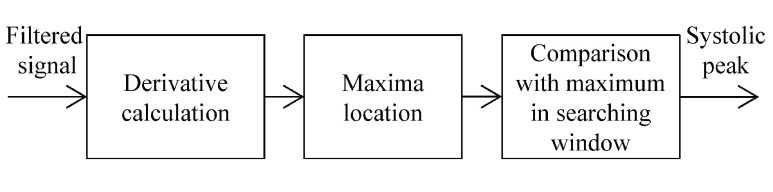
Flowchart for systolic peak detection.

Systolic peaks were detected using local maxima. The flowchart is detailed in Fig. 3. Each candidate maximum point was firstly located using derivative calculation, and then a searching window was employed in attempt to verify whether the maximum was systolic peak or not. The midpoint of searching window was the candidate maximum. If the maximum in the window is equal to the candidate maximum, the candidate point would be defined as systolic peak. Given that the systolic peak is the maximum value in a pulse wave cycle, the length of data points in the window should be more than half of a cycle. After a series of tests, the width of the window was set as 0.6 s.

The trough was defined as the minimum value of two adjacent systolic peaks.

There were 6346 pulse cycles in the selected data. The accuracy of fiducial point detection was over 99.9% with ten systolic peaks and forty-nine troughs detected incorrectly.

#### 3) Baseline Adjustment and Amplitude Normalization

**FIGURE 4.**
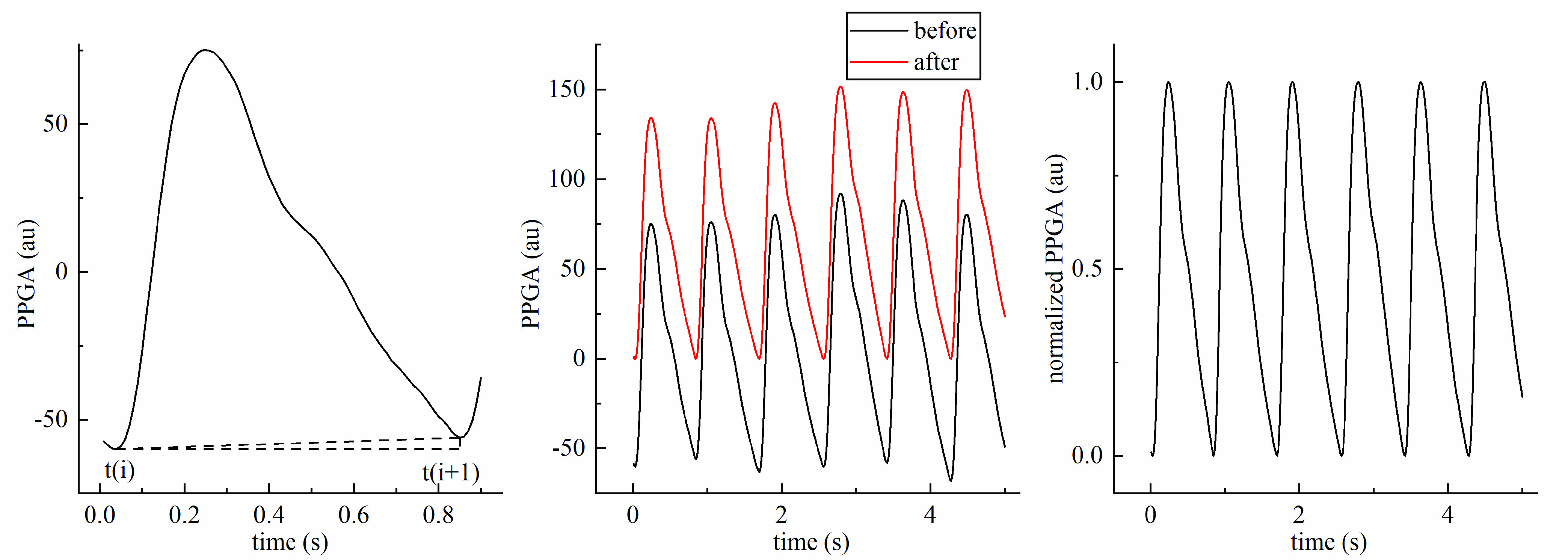
An example of processed photoplethysmographic signal. The left is a diagrammatic sketch of baseline drift removal. The signal in the middle is the selected recording of 5 s before and after baseline drift removal. The right presents the corresponding signal after amplitude normalization.

Interfered by skin depth, skin color, humidity, and ambient light [22], the relative amplitude of PPG varies greatly from person to person. To eliminate the negative impact on amplitude-related features, the normalization of amplitude in each pulse cycle was conducted before feature extraction. The amplitude of each point $(P(j))$ within a pulse cycle was first adjusted to correct the baseline drift:

\begin{align*} k=&\frac {P(t(i+1))-P(t(i))}{t(i+1)-t(i)}, \tag{3}\\ P_{Adjust} (j)=&P(j)-P(t(i))-k\times (j-t(i)), \\&\quad t(i)< j\le t(i+1),\tag{4}\end{align*}

where $P_{Adjust}(j)$ denotes the value of amplitude after baseline drift removal and $t(i)$ denotes the position of troughs. Then each value was divided by its systolic peak amplitude as presented in Fig. 4.

### D. Feature Extraction

**FIGURE 5.**
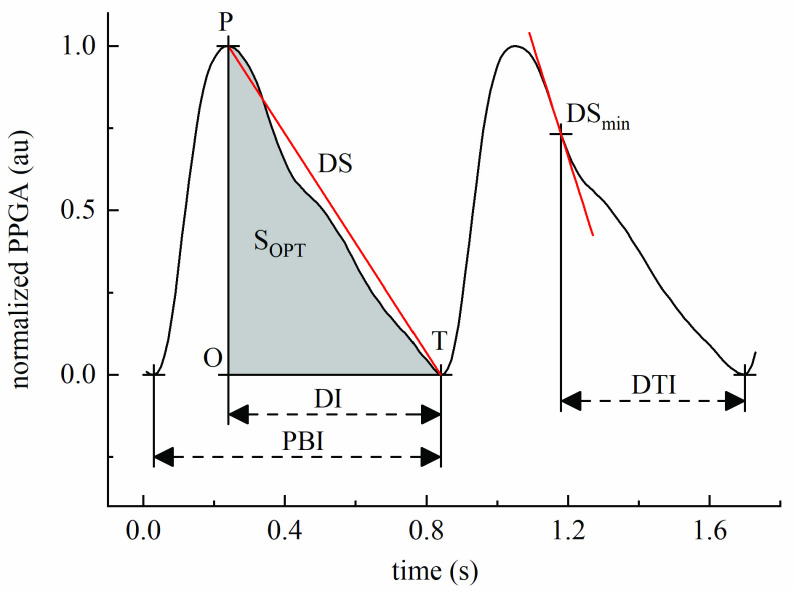
Demonstration of a typical photoplethysmographic signal and its parameters. PBI, pulse beat interval; DI, diastolic interval; DS, diastolic slope; DSmin, the minimum slope during catacrotic phase; DTI, the interval between DSmin and its nearest trough; $\text{S}_{\mathrm {OPT}}$, area under curve of PT.

In order to depict the details of catacrotic phase, five parameters were extracted including diastolic interval (DI), diastolic slope (DS), the minimum slope during catacrotic phase (DSmin), the interval between DSmin and its nearest trough (DTI), and area difference ratio (ADR) defined as:

\begin{equation*} \textrm {ADR}=\frac {S{}_{\Delta OPT}-S{}_{OPT}}{S{}_{\Delta OPT}},\tag{5}\end{equation*}

where $\text{S}_{\mathrm {OPT}}$ is the area under curve of PT and $\text{S}_{\mathrm {OPT}}$ is the area of triangle OPT. Meanwhile, PBI defined as the time interval of the adjacent troughs of PPG was extracted as a reference parameter for its good performance in monitoring nociception [5], [13]. Fig. 5 provides a diagram of these parameters. What should be noted was that the absolute values of DSmin were adopted for subsequent analysis.

The normalization of parameters was carried out via mean value and root mean square (RMS) value [23]:

\begin{equation*} par_{norm} =\frac {par-mean_{pre}}{RMS_{pre}},\tag{6}\end{equation*}

where *mean*$_{pre}$ and *RMS*$_{pre}$ are calculated using the processed data of −70 to −10 s. Subsequently, two values per parameter were derived from normalized parameter to indicate the state before and after stimulation:

1. pre-value: average value of normalized parameter (−70) to (−10) s before stimulus.
2. post-value: average value of normalized parameter (+10) to (+70) s after stimulus.

### E. Statistical Analysis

Sample size calculation was performed using G*power software (version 3.1, Franz Faul, Universitat Kiel, Germany). Owing to the normalization of parameters, the post-value could be regarded as change rate. We assumed that if the difference in change rate of PBI between two groups is equal to or larger than 10%, these two groups are significantly different in terms of PBI. To detect a difference of 0.1 in PBI between two groups with a type I error of $\alpha =0.05$, a power of 1–$\beta =0.8$, and a SD of 0.08, a sample size of 12 in each group would be needed.

All tests were two-tailed with statistical significance defined as $P < 0.05$. The following statistical tests were employed using SPSS (version 25.0, SPSS Inc., Chicago, IL, USA):

1. Kruskal-Wallis test to contrast the distribution of demographic data among three groups.
2. Spearman’s rank correlation test to determine the strength and direction (positive or negative) of the association between two parameters after LMA insertion.
3. Wilcoxon signed-rank test to observe the response of parameters to stimulus at a target concentration of analgesics by comparing pre-values with post-values.
4. Mann-Whitney test to examine the ability of the parameters to discriminate between two different concentrations during nonnoxious and noxious period.
5. Prediction probability ($\text{P}_{\mathrm {K}}$) was computed using PKMACRO [24] to test the potential of parameters to grade the level of analgesia, i.e., to assess whether post-values of parameters compose a concordance with the increasing remifentanil concentrations in the same or opposite direction.

## III. Results

All 45 patients (15 patients in each group) were included in the final analysis. There were no significant differences among three groups regarding demographic characteristics of patients such as age, height, weight, and BMI (Table 3).

**TABLE 3:** Demographic Characteristics of Patients

	Three groups of remifentanil target effect-compartment concentration			All patients
	N=15, 0ng/ml, R0	N=15, 1ng/ml, R1	N=15, 3ng/ml, R3	N=45
Age (yr)	48 (41–52)	42 (36–48)	46 (34–49)	46 (36–50)
Height (cm)	160 (158–164)	160 (157–163)	160 (158–163)	160 (158–163)
Weight (kg)	58.0 (56.0–62.0)	57.0 (54.0–62.0)	56.0 (52.0–60.0)	57.0 (54.0–61.0)
BMI (kg/$\text{m}^{2}$)	23.05 (20.96–24.14)	23.05 (21.09–23.73)	21.88 (20.43–23.11)	22.66 (20.89–23.73)

Data are median ($25^{\mathrm {th}}$–$75^{\mathrm {th}}$ percentile).

### A. Correlation Between Parameters

Parameters except ADR were correlated significantly with each other (Table 4). A comparatively strong dependence was discovered with respect to three temporal-related parameters, namely PBI, DTI, and DI. The largest Spearman’s rank correlation coefficient was obtained when DI and DS were analyzed.

**TABLE 4:** Spearman’s Rank Correlation Coefficient for Post-Values of Parameters

		PBI	DI	DS	DSmin	DTI	ADR
Spearman’s rank correlation coefficient	PBI	1.000	0.933^**^	−0.914^**^	−0.547^**^	0.951^**^	0.178
	DI	0.933^**^	1.000	−0.989^**^	−0.597^**^	0.952^**^	0.108
	DS	−0.914^**^	−0.989^**^	1.000	0.614^**^	−0.947^**^	−0.120
	DSmin	−0.547^**^	−0.597^**^	0.614^**^	1.000	−0.543^**^	0.119
	DTI	0.951^**^	0.952^**^	−0.947^**^	−0.543^**^	1.000	0.435
	ADR	0.178	0.108	−0.120	0.119	0.435	1.000

^**^ ${P}< 0.01$.

### B. Responses to Stimulus Under Different Remifentanil Target Concentrations

**FIGURE 6.**
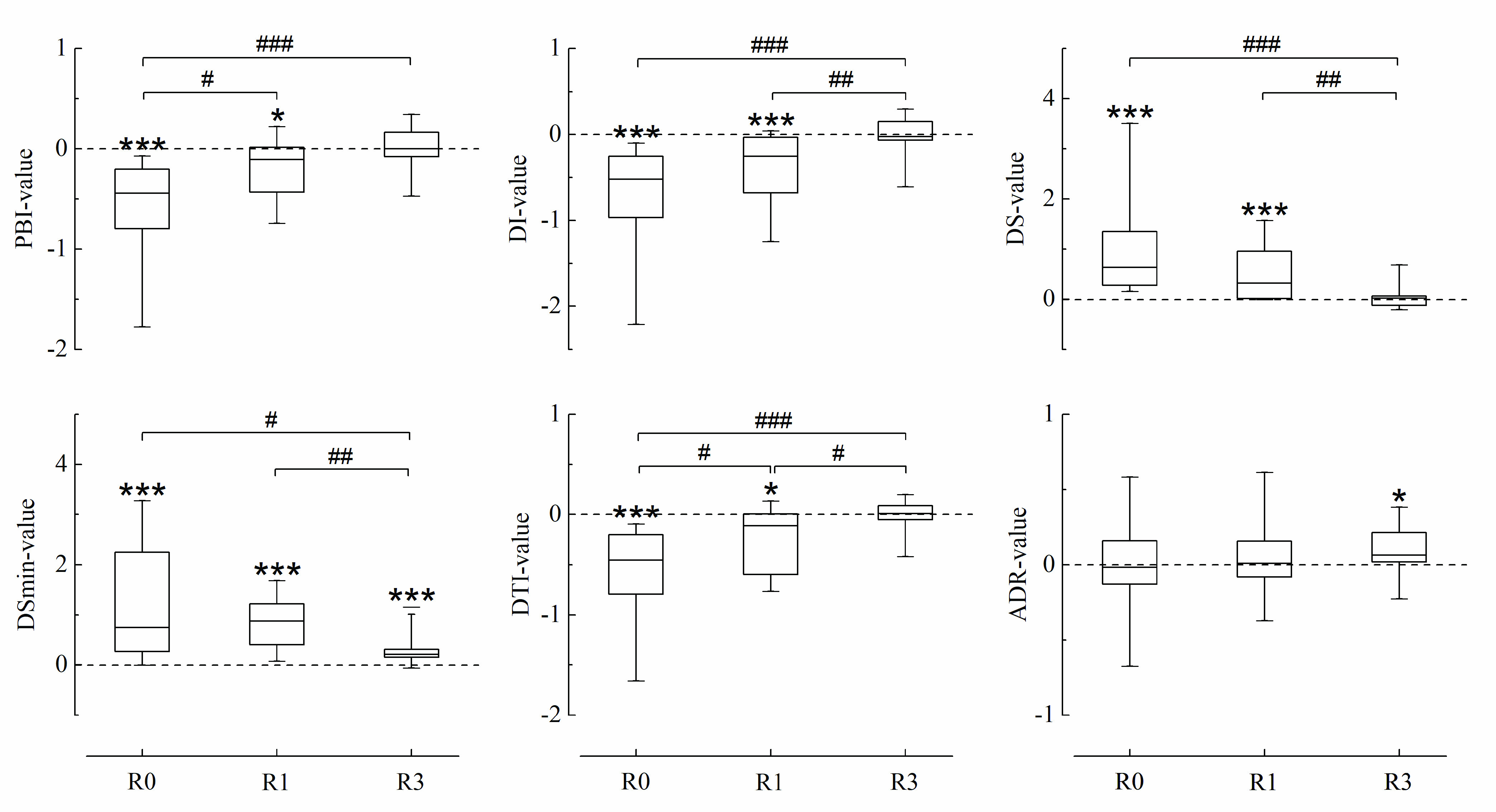
Parameters after LMA insertion at remifentanil effect-compartment concentrations of 0, 1, and 3 ng/ml (R0, R1, and R3, respectively). PBI, pulse beat interval; DI, diastolic interval; DS, diastolic slope; DSmin, the minimum slope during catacrotic phase; DTI, the interval between DSmin and its nearest trough; ADR, area difference ratio. Black dotted lines represent the states of parameters before stimulation. Data are median, interquartile range, and range. *Wilcoxon signed-rank test, $^\ast P< 0.05$, $^{\ast \ast }P< 0.01, ^{\ast \ast \ast }P< 0.001$; #Mann-Whitney test, #$P < 0.05$, ##$P < 0.01$, ###$P < 0.001$.

There were no significant discrepancies demonstrated in pre-values of all parameters among three groups. The effect of stimulus on parameters under various analgesic concentrations is displayed in Fig. 6. LMA insertion elicited a remarkable decrease in temporal-related parameters, and an increase in slope-related parameters including DS and DSmin during Ce_remi_ of 0 and 1 ng/ml whereas no obvious variation was captured in ADR. The consistent changes induced by stimulus were only observed in DSmin.

It was apparent that five parameters exclusive of ADR were statistically dependent on the tested Ce_remi_ in varying degrees after stimulus. ADR was approximately unchanged whereas other parameters varied progressively in a constant direction with the deepening of analgesia. The discrimination between R0 and R3 could be identified by all the parameters except ADR. PBI and DTI could differentiate R0 from R1. Only DTI successfully graded the response to the change of concentration from 0 to 1 ng/ml, and then to 3 ng/ml.

### C. Prediction Probability Analysis

Compared to PBI ($\text{P}_{\mathrm {K}} =0.796$), three parameters of DI, DS, and DTI showed a better consistence with the level of anti-nociceptive medication (Table 5). DI yielded the largest $\text{P}_{\mathrm {K}}$ value, 0.825.

**TABLE 5:** Prediction Probability ($\text{P}_{\mathrm{K}}$) Values for Post-Values of Parameters

	PBI	DI	DS	DSmin	DTI	ADR
$\text{P}_{\mathrm {K}}$	0.796 (0.045)	0.825 (0.045)	0.822 (0.044)	0.689 (0.073)	0.822 (0.041)	0.627 (0.069)

Data are $\text{P}_{\mathrm {K}}$-values (standard error).

**FIGURE 7.**
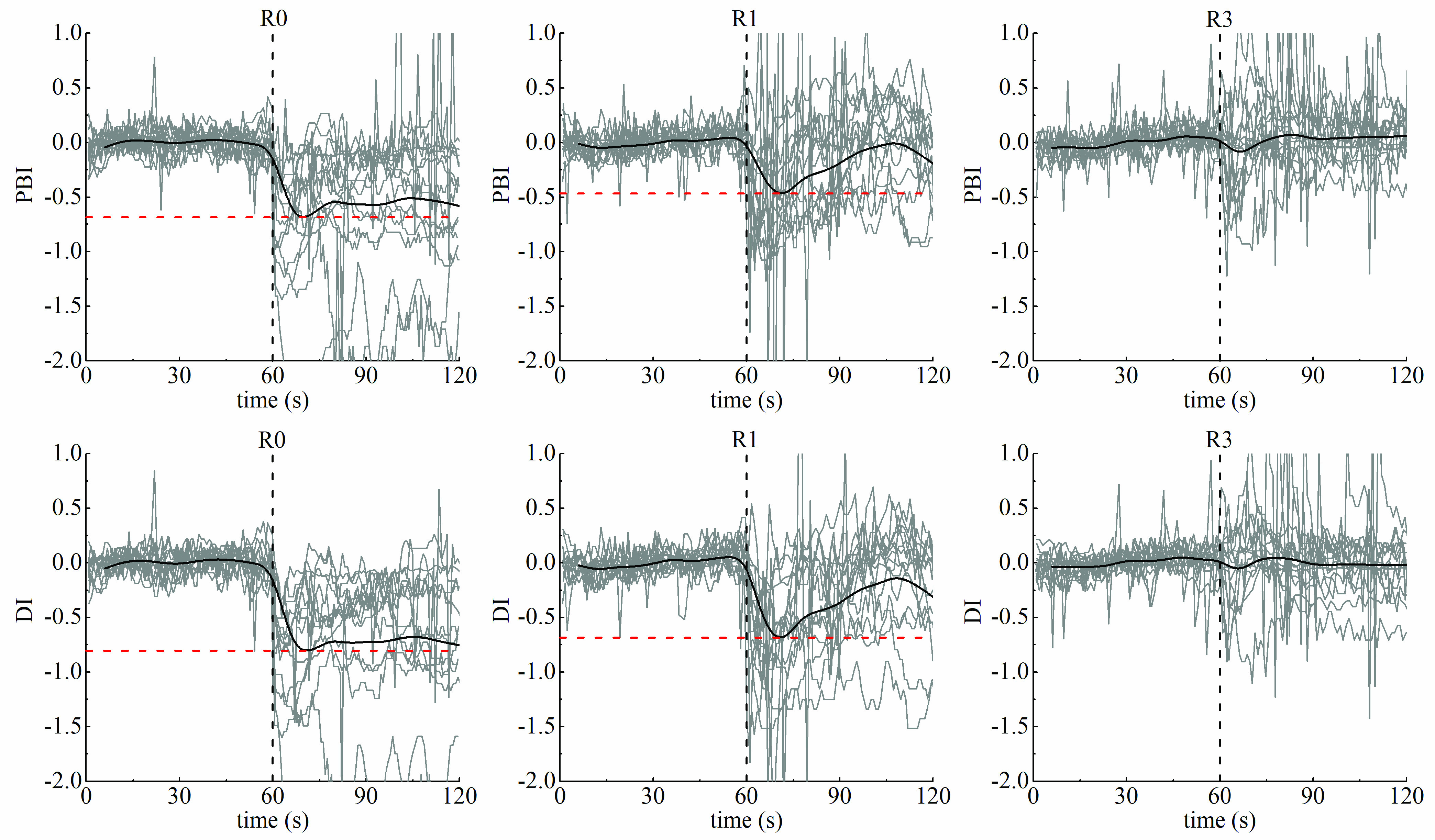
Plots of individual time courses (gray lines) and mean time course (dark lines) of pulse beat interval (PBI) and diastolic interval (DI) in three groups during 60 s before and after stimulation (black dotted line). Red dotted lines represent the minimum values of the mean time courses.

Take a close look at individual plots and trends of PBI and DI (Fig. 7). The trends of PBI and DI were quite similar. The values of them were both kept at a relatively steady state during the first 60 s. In the presence of stimulation, they were dramatically altered when no or a low concentration was given, whereas no conspicuous decrease existed under Ce_remi_ of 3 ng/ml. According to the mean time courses, in contrast with the values retained at a specific level in R0, the values in R1 gradually increased after reaching their minima.

## IV. Discussion

Five parameters were extracted to portray the catacrotic phase of PPG in time domain and to explore the relationship with the balance of NAN. The responses of parameters to clinical stimulus under various concentrations of remifentanil were compared with PBI’s.

Although different study protocols have been proposed to examine the correlation between measures and balance of NAN, for example, some researchers [25]–[27] applied tetanic stimulus at varying concentrations of remifentanil in one person whereas Bonhomme *et al.* [28], Rantanen *et al.* [29], and our study assigned patients to several groups and observed the response of each patient to intraoperative stimulation at only one target concentration of analgesics, the essence of study design is identical, that is, keeping hypnotic level and nociception level constant when altering the level of analgesia. It is well known that general anesthesia is mainly consisted of hypnosis and analgesia; therefore, the prerequisite for analyzing analgesia during general anesthesia is to adjust the level of hypnosis to an adequate and appropriate state. SE is a well-accepted index to monitor hypnosis, and SE-guided anesthesia has been proved to reduce the probability of consciousness during surgery [30]–[32]. The range of 40 < SE < 60, which has been recommended as an interval of “clinically meaningful anesthesia”, was chosen in this study for guiding propofol titration [32], [33]. Meanwhile, we designated the same anesthesiologist to complete LMA insertion for all enrolled patients to minimize the discrepancy and achieve a comparable nociception level.

Methodologically, our analysis was launched using the average values of parameters in the window before and after the stimulus. The duration of the window and the selected response descriptor could affect outcomes. The window of 60 s before (−70 to −10 s) and after (+10 to +70 s) stimulus was suitable for evaluation considering that LMA insertion lasts no more than 30 s, and longer time, such as two minutes [29], [34], would blunt the response with the recovery of physiological parameters. Moreover, the discarded data (−10 to +10 s) could separate the data before and after the event clearly without losing the crucial information generated by stimulus. Both maximum and average value have been used to describe the reactions to stimulus [25]–[28]. Maximum could preferably characterize some salient features which increase with the existence of noxious event, but it is vulnerable to outliers and could not explain the decreased features felicitously. In contrast, average value is advantageous to detect the sustained responses, and more likely to provide relevant estimation of parameters with diverse variation trends in this study.

The normalization of PPG amplitude and parameters were conducted for eliminating the inter- and intra-subject variability of parameters. Amplitude normalization plays an important role in diminishing the influence caused by skin condition and surroundings and giving prominence to the temporal-related features. It is precisely because of the eradication of differences in PPG systolic peak amplitude that the Spearman’s rank correlation coefficient between DI and DS was the largest. With regard to the normalization of values before and after event, there are two considerations. On the one hand, remifentanil could disturb heart rate whether noxious stimulus was applied or not [35], [36]; on the other hand there is a strong relationship between heart rate and PPG time-related parameters like PBI [21], [37]. Accordingly, the accuracy in the assessment of responses to stimulus would be reduced if without removing the effect of analgesics on parameters during nonnoxious period. As expected, there was no obvious distinction among three groups in terms of pre-values of all normalized parameters. The normalization of parameters was performed by RMS and mean values in this study, rather than subtracting or dividing average value to calculate change rate [15], [38]. These methods have been compared before the study and the parameters processed using RMS and mean value showed a better concordance with concentrations.

The results revealed that only DSmin consistently detected the stimulus. DSmin indicates the fastest rate of the decline in blood volume. Although it was reasonable that there were no statistical differences discovered in other parameters before and after stimulation based on the fact that Ce_remi_ of 3 ng/ml was sufficient for LMA insertion, this might also imply that noxious events could deform the shape of PPG irrespective of analgesics concentrations. Regretfully, the predictive power of DSmin on the analgesic drug level was poor ($\text{P}_{\mathrm {K}} =0.689$).

The parameters of PBI, DI, DS, and DTI possessed a strong predictive power on the level of analgesia. It is also noteworthy that these four parameters were strongly correlated with each other; therefore, it is not advisable to choose two or more from them for the further development of multivariate index. Additionally, the $\text{P}_{\mathrm {K}}$ value of PBI was relatively smaller by comparison with DI and DTI, it might be conjectured that the synthetic effect of noxious event and analgesics on catacrotic phase is more significant than that on anacrotic phase. The variation that the trends of PBI and DI showed a steep descent after stimulation suggests that the insufficient analgesia is perceived by autonomic reactions of increases in heart rate, and the following alteration that a small fluctuation when no remifentanil was administrated whereas a clear upward trend under Ce_remi_ of 1 ng/ml corroborates the counteraction of analgesics on the nociception.

There are some limitations in this study. First, to ascertain how feasible the parameters in monitoring the balance of NAN during general anesthesia might be, more patients need to be included. Second, clinically, the intensity of stimulation varies during the surgery, yet we have only investigated the response to LMA insertion. More types of noxious events should be studied and compared. Third, all patients in this study were female and the association between parameters and analgesic concentrations in male patients should be further verified considering the gender difference in analgesia [39].

## V. Conclusion

This study extracted five parameters from catacrotic phase of PPG and compared them with PBI in grading the level of analgesia. The features including DI, DS, and DTI could provide a better potential to qualify the balance of NAN. Further validation studies are required to elucidate the feasibility.
